# Optimization and Economic Impact of Expedited Minimally Invasive Parafascicular Surgery (MIPS) Protocol for Spontaneous Intraparenchymal Hemorrhage

**DOI:** 10.7759/cureus.81234

**Published:** 2025-03-26

**Authors:** Mayur S Patel, Arianna Carfora, Kathleen S Botterbush, Jorge F Urquiaga, Jeroen R Coppens

**Affiliations:** 1 Neurological Surgery, Saint Louis University School of Medicine, St. Louis, USA

**Keywords:** cost reduction, economic burden, intracranial hemorrhage, minimally invasive, protocol

## Abstract

Background

Minimally invasive parafascicular surgery (MIPS) for evacuating intracerebral hemorrhage (ICH) has proven to be an effective treatment compared to medical management. At our academic center, we have adopted a strategy of early surgery (<12 hours) and aimed to assess its impact on patients undergoing MIPS from an economic standpoint. This study introduces an innovative preoperative protocol to reduce costs and improve efficiency in the healthcare setting for patients undergoing ICH evacuation.

Methods

A retrospective review was conducted on patients who underwent MIPS for spontaneous ICH evacuation between 2014 and 2017. The patients were stratified into two groups: expedited versus control and early versus late operation. The expedited protocol involved using either computed tomography angiography (CTA) or a stereotactic head CT for guidance during the operation.

Results

Nine patients were included in the expedited protocol group, where they were taken from the emergency department (ED) for CT and CTA, followed by a surgical decision, and then directly to the operating room (OR) from the imaging center. Nine patients were included in the control group, where they were taken from the ED for CT and CTA and returned to the ED, followed by a surgical decision, then to the imaging center for a stereotactic CTH for intraoperative navigation, and then to the OR. Additionally, eleven patients were in the early operation group, and seven were in the late operation group. The mean time from ED admission to surgery was 8.2 hours for the early operation group and 62.2 hours for the late operation group (p = 0.10). The control group had 38 preoperative scans, while the expedited group had 17. The mean preoperative imaging cost decreased from $2,039 in the control to $1,003 in the expedited group (p = 0.004). Similarly, the mean preoperative imaging cost was $2,061 for the late operation group and $1,162 for the early operation group, respectively (p = 0.02). There was a 15% decrease in the postoperative hospital stay cost per patient (p > 0.05).

Conclusion

Patients undergoing an expedited preoperative protocol and early surgery experienced a statistically significant reduction in preoperative costs and a shorter time between ED admission and surgery. The expedited protocol may provide economic relief for patients undergoing MIPS without compromising outcomes.

## Introduction

Spontaneous intracranial hemorrhage (ICH) is a significant cause of disability and mortality, with studies reporting as low as a 38% survival rate in the first year [1,2]. Both medical and surgical techniques have been developed for the treatment of spontaneous supratentorial ICH; however, its optimal treatment remains unknown [3]. Prior studies did not demonstrate a universal benefit of surgery over medical management, though subgroup analyses suggested potential advantages in select cases [4,5]. Minimally invasive surgical techniques were developed after those failures, with the two most recent techniques being minimally invasive parafascicular surgery (MIPS) and stereotactic intracerebral hemorrhage underwater blood aspiration (SCUBA). The use of the SCUBA technique led to the randomized MIND trial, which was stopped early [6-8]. Similarly, the use of the MIPS technique led to the ENRICH trial, which studied the use of a MIPS within 24 hours of the last known well versus medical management [6-8].

Studies have reported that spontaneous ICH has a mean of 9.5 disability-adjusted life years, 5.72 average years of life lost, and a mean of 3.74 years of life lived with disability [9]. Additionally, secondary injury from spontaneous intraparenchymal ICH, such as hematoma expansion, intraventricular hemorrhage, and hemorrhage-associated edema, further increases the disease burden [9]. Complications from ICH, including hydrocephalus, seizures, infections, and edema, also significantly contribute to the cumulative postoperative costs [10].

In addition to its high mortality rate, ICH carries a substantial economic burden for patients and providers alike. In an economic analysis of the financial costs associated with ICH in the United States, Bedaiwi et al. described a $49,020 increase in per capita hospital cost (from $63,813 to $112,833) for ICH management from 2006 to 2014, which is equivalent to $65,134 in 2024 [11,12]. Furthermore, economic burden is enhanced by the high presence of medical complications in this patient population [13].

Previous case series studies have demonstrated that MIPS significantly reduces 30-day mortality compared to medical management [2,14]. This is further confirmed by class I evidence from the ENRICH trial. The trial showed that among patients who underwent surgery within 24 hours after an acute intracerebral hemorrhage, minimally invasive hematoma evacuation resulted in better functional outcomes at 180 days [8]. In addition to its clinical benefits, MIPS has exhibited a reduced per capita hospital financial burden when compared with traditional craniotomies in preliminary studies [12]. At our institution, we have utilized the MIPS technique in selected cases of spontaneous supratentorial ICH since 2014. Our protocol was revised in 2017 to optimize preoperative management for MIPS candidates, aiming to reduce the time from onset to surgery.

While the ENRICH trial demonstrated improved functional outcomes with MIPS, data on its economic impact remain limited. This study aims to evaluate whether an expedited preoperative protocol can optimize costs and efficiency in ICH management. Additionally, our goal is to introduce novel techniques that reduce costs and improve efficiency in the healthcare setting for patients undergoing ICH evacuation.

## Materials and methods

Study design

We conducted a single-center retrospective cohort study on patients who underwent MIPS for the evacuation of spontaneous basal ganglia and lobar ICH between 2014 and 2017. Ethical approval was obtained from our institutional review board. Patients aged 18-80 years with a Glasgow Coma Scale (GCS) score of 5-14 were included. Eligible patients also had an ICH volume of 30-80 mL with a modified Rankin scale (mRS) score of 0-1 prior to the ICH. Only patients with spontaneous ICH in the deep cortical structures or cortex were included. Additionally, patients must be surgical candidates within 24 hours of ED presentation. Exclusion criteria included age younger than 18 or older than 80 years, an ICH score of IV, intraventricular hemorrhage affecting more than 50% of the ventricular system, mRS > 1 prior to hemorrhage, and irreversible coagulopathies. 

Patients were stratified into two groups: an expedited and a traditional control group. The expedited group involved using computed tomography angiography (CTA) for facial registration in the operating room (OR). The control group included patients who received separate CTA, followed by a dedicated stereotactic computed tomography (CT) scan with fiducials before surgery. Patients were also stratified based on undergoing an operation before (“early operation”) or after 12 hours after admission (“late operation group”). Only patients who underwent evacuation prior to our enrollment in the ENRICH trial were included in this study [15]. The control group included patients who presented to the ED and were evaluated by a noncontrast head CT (NCCT) followed by CTA. Patients were returned to the ED before a surgical decision was made. If they were candidates for MIPS, fiducials were placed, followed by an additional NCCT, before proceeding to the OR.

Patient information

Demographic variables, ICH score, length of the operation, and GCS score were collected. Imaging data included preoperative scans (NCCT at our institution), the number of NCCT obtained at an outside hospital, magnetic resonance imaging (MRI), MR angiogram (MRA), CTA, NCCT using a stereotactic protocol with facial markers, and postoperative NCCT. Postoperative outcomes were assessed by the number of days spent in the ICU with and without mechanical ventilation and the length of inpatient stay. Our primary outcomes were preoperative costs, the number of preoperative images, and postoperative costs. Our secondary outcomes included the total number of postoperative scans, the number of days spent in the ICU (both ventilated and non-ventilated), the total cost of hospital stay, and discharge location. Changes in preoperative and postoperative GCS and ICH scores were compared to assess the efficacy of the treatment.

Expedited protocol

**Figure 1:**
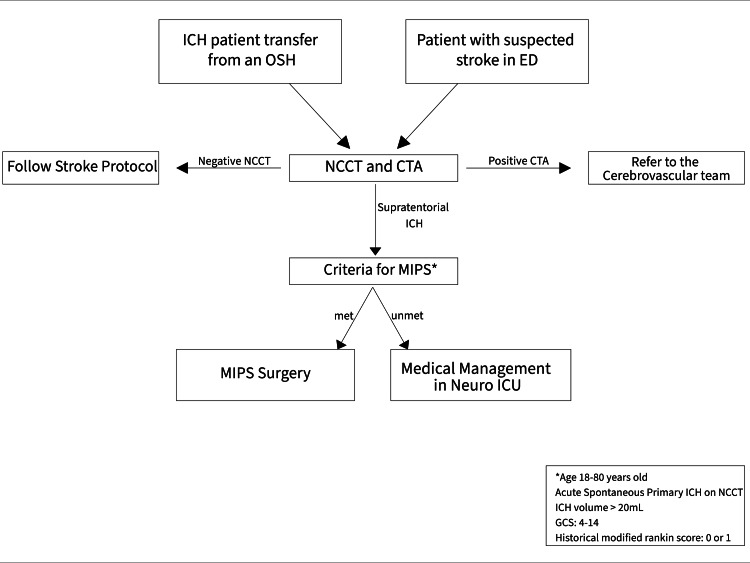
Flowchart of the decision-making process for selecting candidates for minimally invasive parafascicular surgery technique utilizing the expedited protocol developed at our institution. ICH: intracerebral hemorrhage, OSH: outside hospital, ED: emergency department, NCCT: noncontrast CT scan of the head, CTA: computed tomography angiography, MIPS: minimally invasive parafascicular surgery, GCS: Glasgow Coma Scale, ICU: intensive care unit.

The expedited protocol is a preoperative protocol developed at our center to reduce hospital costs, improve efficiency, and optimize patient outcomes. However, the crux of this protocol is to minimize the use of excessive preoperative brain imaging and patient transport without compromising outcomes (Figure 1).

**Figure 2:**
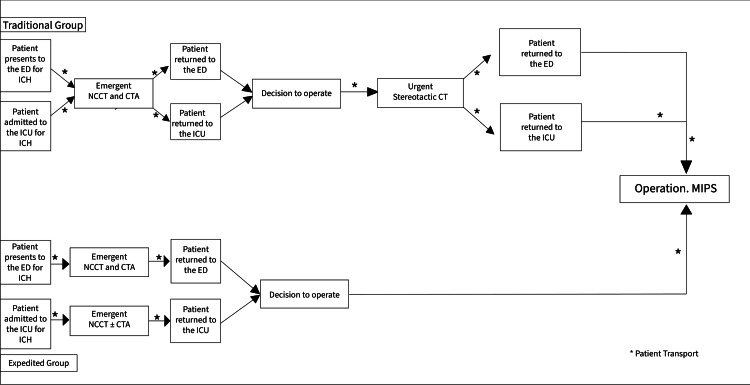
Flowchart of the expedited preoperative protocol developed at our institution for patients undergoing MIPS for intracranial hemorrhage evacuation compared to a traditional preoperative protocol. NCCT: noncontrast CT scan of the head, CTA: computed tomography angiography, ICU: intensive care unit, ED: emergency department, ICH: intracranial hemorrhage. Asterisk (*) indicates patient transport to the next location.

Under the expedited protocol, patients were taken from the ED to have the NCCT and CTA. A surgical decision was made, and patients were taken directly to the OR from the imaging center or the ED (if the patient had returned to the ED) (Figure 2).

Cost analysis

The total professional cost for imaging was calculated based on the total relative value units (RVUs), total geographic practice cost index (GPCI), and conversion factor (CF) value based on the current procedural terminology (CPT) codes for the type of scan [16]. This was added to the total hospital cost associated with each CPT code [16,17]. Using this, the total preoperative cost of images and postoperative NCCT cost was calculated. Because the study was conducted from 2014 to 2017, costs were adjusted from the date of the study end via the CPI inflation calculator from the United States Bureau of Labor Statistics. The reference date for cost adjustment was August 2024.

Costs associated with the number of days in the ICU while mechanically ventilated were calculated using previously published data ($13,981 for the first day, $6,212 for the second day, and $5,139 for more than three days). Costs associated with the number of days in the ICU without ventilation were also calculated ($8,635 for the first day, $4,528 for the second day, and $4,124 for more than three days) [18]. The cost of the inpatient stay per day was $3,377 [19]. The same costs per day and per resource were assigned to those days and resources irrespective of the year of presentation. Using these values, the total cost of postoperative hospital stay cost was calculated. The costs were based on 2020-2021 and then adjusted to August 2024 accounting for inflation. Patients who died postoperatively were excluded from postoperative cost calculation and statistical analysis as this affects the mean number of postoperative ICU and inpatient stay days.

The true cost to the hospital, charges to the patient, and charges to insurance companies were not available, so standard Medicare costs using RVU and CPT codes were used. The cost of transfers from outside hospital systems was also not publicly available. Prices and costs at our institution may vary significantly from those at other hospital systems. Many essential hospital costs may have not been accounted for, such as costs associated with medication, usage of hospital supplies, food, and staffing.

Statistical analysis

Descriptive statistics were performed to compare baseline demographic, clinical, and surgical characteristics between groups. Mean and standard deviation were used for numeric outcomes, and frequency and percentages were used for categorical outcomes. The Shapiro-Wilk test was used to test for the normality of the data. Independent samples t-tests and chi-square tests were used to compare cost outcomes when the data were normally distributed. As our n was low, nonparametric, Wilcoxon-rank sums, and Fisher’s exact tests were utilized in this study. Cohen’s d was used to calculate effect size. A p-value of <0.05 was considered statistically significant. All statistical analyses were performed using R software, version 4.1.0 (R Foundation for Statistical Computing, Vienna, Austria). 

## Results

Expedited group versus control protocol group

A total of 18 patients (nine expedited and nine control) were studied. Demographic, clinical, and operative characteristics are reported in Table 1.

**Table 1:** Demographics and preoperative and postoperative outcomes of patients stratified by the preoperative protocol (control vs expedited). ICH: intracranial hemorrhage, GCS: Glasgow Coma Scale, CT: computed tomography, CTA: computed tomography angiography, MRI: magnetic resonance imaging, MRA: magnetic resonance angiogram, ICU: intensive care unit. *p < 0.05.

Characteristics	Traditional protocol	Expedited protocol	p-value	Degrees of freedom	Effect size
No. of patients	9	9			
Age (years)					
Mean ± SD	61.4 ± 9	54.8 ± 13.4	0.36		
Gender			0.63	1	0.11
Male	5 (56%)	6 (67%)			
Female	4 (44%)	3 (33%)			
Mean ICH score					
Change	0.3 ± 0.7	0.1 ± 0.3	0.3		
Mean GCS					
Preoperative GCS	8.25 ± 2.7	8.6 ± 3.7	0.62		
Postoperative GCS	8.4 ± 1.9	8.2 ± 2.0	0.92		
Change	0.56 ± 2.74	0.67 ± 2.54	0.86		
Time to operation (hours)					
Mean ± SD	62.2 ± 95.1	8.3 ± 8.7	0.1		
Length of operation (minutes)					
Mean ± SD	135.2 ± 29.3	162.7 ± 60.5	0.4		
Number of preoperative scans			0.71	4	0.35
Head CT without contrast	20	8			
Head and neck CTA	8	6			
Brain MRI without contrast	1	1			
Brain MRA	1	0			
Stereotactic head CT	8	2			
Total number of scans	38	17	0.11		
Preoperative imaging cost	2,039.21 ± 920.11	1,003.30 ± 228.37	0.004*		
Postoperative course					
Number of days on ventilator	7.8 ± 5.5	6.2 ± 5.5	0.08		
Number of days in the ICU	11.8 ± 7	11.2 ± 6.6	0.28		
Mean postoperative stay cost when ready for discharge	107,399.80 ± 28,830.51	85,945.77 ± 25,291.62	0.14		
Mean postoperative stay cost when actually discharged	110,766.80 ± 28,828.68	92,396.80 ± 25,648.21	0.22		
Total postoperative care cost (cost of stay + postoperative imaging)	111,707.60 ± 28,996.98	95,247.39 ± 24,563.23	0.24		
Discharge location			0.88	3	0.19
Acute rehabilitation facility	4 (44%)	4 (44%)			
Supervised nursing facility	2 (22%)	2 (22%)			
Hospice	2 (22%)	1 (11%)			
Death	1 (11%)	2 (22%)			
Length of stay					
Mean ± SD	20.8 ± 8.1	21.3 ± 14.4	0.56		

There were five (56%) male patients in the control group and six (67%) in the expedited group. The mean age was 61.4 and 54.8 years in the control and expedited groups, respectively (p = 0.36, Cohen’s d = 0.58, 95% CI 52.77-63.46). The mean change in ICH score between ED admission and immediately before the surgery was 0.3 and 0.1 in the control and expedited groups, respectively (p = 0.3, Cohen’s d = 0.40, 95% CI -0.03 to -0.47). Furthermore, the change in preoperative and postoperative GCS scores was 0.3 in the control group and 0.7 in the expedited group (p = 0.86, Cohen’s d = 0.04, 95% CI -1.09 to -1.65).

The mean time for patients to undergo MIPS in the control group was 62.2 hours and 8.2 hours in the expedited group (p = 0.10, Cohen’s d = 0.80, 95% CI 2.40-68.11). Additionally, the mean preoperative imaging cost was $2,039 and $1,003 for the control and expedited groups, respectively (p = 0.004, Cohen’s d = 1.55, 95% CI $1,132-$1,909). There were no intraoperative complications in both groups. In the expedited protocol group, four patients developed pneumonia and one developed clinical seizures, which resolved during the stay. In the control group, two patients developed pneumonia, one developed a urinary tract infection, one was found to have atrial fibrillation, and one patient developed superficial vein thrombosis.

Patients in the control group spent an average of 7.8 days on a mechanical ventilator and an additional 11.8 days after extubation postoperatively in the ICU. Patients in the expedited group spent an average of 6.2 days on a mechanical ventilator and a further 11.2 days after extubation in the ICU (while intubated: p = 0.08, Cohen’s d = 0.28, 95% CI 4.51-9.49; while extubated: p = 0.28, Cohen’s d = 0.08, 95% CI 8.44-14.56). Lastly, the postoperative stay cost (control $110,766 vs expedited $85,945; p = 0.22, Cohen’s d = 0.67, 95% CI $89,230-$115,158) and the total postoperative care cost, which includes the mean postoperative stay cost and postoperative imaging cost (control $111,707 vs expedited $95,247), were similar (p = 0.24, Cohen’s d = 0.67, 95% CI $86,572-$107,931).

Four patients in both groups were discharged to an acute rehabilitation center. Furthermore, one patient in the control group and two patients in the expedited group died postoperatively in the ICU. The mean length of stay in the hospital was 20.8 and 21.3 days for the control and expedited groups, respectively (p = 0.56, Cohen’s d = 0.07, 95% CI 15.65-26.15).

Early operation group versus late operation group

There were 11 patients in the early surgery group and 7 in the late surgery group. The mean difference in ICH score between ED admission and before the operation and the change in preoperative and postoperative GCS scores were not significant. Additionally, the mean preoperative imaging cost was $2,061 and $1,162 for the late operation group and early operation group, respectively (p = 0.02, Cohen’s d = 1.11, 95% CI $1,073-$1,924). There were no intraoperative complications in both groups.

Patients in the late surgery group spent an average of 9.4 days on a mechanical ventilator and an additional 13.8 days after extubation in the ICU after the surgery, and patients in the early operation group spent an average of 5.5 days on a mechanical ventilator and a further 10.4 days after extubation in the ICU (while intubated: p = 0.08, Cohen’s d = 0.77, 95% CI 4.46-9.54; while extubated: p = 0.27, Cohen’s d = 0.46, 95% CI 8,79-14.21). The mean number of days spent on the inpatient floor was 3.4 and 11.9 for the late and early operation groups, respectively (p = 0.06, Cohen’s d = 1.18, 95% CI 4.46-12.76) (Table 2).

**Table 2:** Demographics and preoperative and postoperative outcomes of patients who underwent the operation within 12 hours of ED admission (early operation group vs late operation group). ICH: intracranial hemorrhage, GCS: Glasgow Coma Scale, ICU: intensive care unit. *p < 0.05.

Characteristics	Operation time ≤ 12 hours	Operation time > 12 hours	p-value	Degrees of freedom	Effect size
No. of patients	11	7			
Age (years)					
Mean ± SD	55.9 ± 11.5	61.6 ± 11.6	0.36		
Gender			0.66	1	0.1
Male	7 (66%)	6 (55%)			
Female	4 (34%)	5 (45%)			
Mean ICH score					
Change	0.1 ± 0.3	0.4 ± 0.8	0.3		
Mean GCS					
Preoperative GCS	9.4 ± 3.1	8.1 ± 2.1	0.1		
Postoperative GCS	7.5 ± 3	8.5 ± 1.9	0.89		
Change	-1.3 ± 3	1.3 ± 2.3	0.11		
Preoperative course					
Preoperative imaging cost	1,162.31 ± 355.56	2,061.19 ± 1,091.69	0.02*		
Postoperative course					
Number of days on ventilator	5.5 ± 5.1	9.4 ± 5.3	0.08		
Number of days in the ICU	10.4 ± 2.6	13.3 ± 4.8	0.27		
Number of days on the inpatient floor	11.9 ± 9.6	3.4 ± 3.4	0.06		
Postoperative imaging cost	964.08 ± 547.74	107,399.80 ± 28,830.51	0.23		
Mean postoperative stay cost when ready for discharge	95,945.77 ± 25,291.62	2,061.19 ± 1,091.69	0.56		
Mean postoperative stay cost when actually for discharge	98,118.69 ± 28,742.57	108,307.93 ± 28,431.36	0.51		
Total postoperative care cost	99,251.09 ± 30,910.85	109,618.70 ± 28,740.76	0.53		
Discharge location			0.65	4	0.3
Acute rehabilitation facility	5 (45%)	2 (18%)			
Supervised nursing facility	3 (27%)	1 (9%)			
Long-term acute care facility	0	0			
Hospice	1 (9%)	2 (18%)			
Death	2 (18%)	1 (9%)			
Length of stay					
Mean ± SD	21.2 ± 13.9	20.6 ± 6.5	0.56		

Of the deaths, two occurred in the early surgery group and one in the late surgery group. There were no repeat surgeries or hematoma reaccumulation that required further procedures in either group. 

## Discussion

ICH carries a higher risk for morbidity and mortality when compared to ischemic stroke. Early deterioration is common in ICH due to an expanding hematoma and/or perihematomal edema [20]. The timing of surgery is still not clearly defined in spontaneous ICH. Still, some data suggest that early management of ICH is highly recommended to reduce both short- and long-term complications [21]. At our institution, we have utilized the MIPS technique for hematoma evacuation since 2014, and our early studies suggest some potential clinical benefits when compared with traditional craniotomies [2,14]. As costs associated with ICH management have increased significantly, we aimed to improve the efficiency of the preoperative MIPS protocol to simultaneously improve efficiency for ICH evacuation.

We developed an expedited protocol that uses either the CTA or a stereotactic NCCT for intraoperative navigation. This eliminates the need for patients to undergo multiple trips between the ED and the radiology areas and reduces the number of imaging sessions required. Furthermore, this reduces the time it takes to decide if surgery is an appropriate intervention for a patient. This is the first retrospective cohort study suggesting a preoperative imaging protocol and assessing the cost burden of the protocol for early intervention in patients undergoing MIPS.

Additionally, it is worth mentioning that although there was no statistically significant difference in age between the expedited and control groups, younger patients may tend to have better outcomes postoperatively. In our study, age was not a determining factor in terms of outcomes. 

Cost outcomes

Based on our observations, the expedited protocol reduced the number of scans that patients underwent preoperatively, from 38 scans in patients who did not undergo the expedited protocol to 17 scans in patients who did (p = 0.11). This significantly decreased the preoperative imaging costs from an average of $2,039 and $1,003 between the two groups (p = 0.004).

Additionally, the expedited protocol reduced the time patients spent waiting to undergo surgery from an average of 62 to 8 hours (p = 0.10). Given the low sample size, this result is nonsignificant but shows a markedly reduced preoperative time spent in the ED. Theoretically, this would also reduce the preoperative hospital stay and ED costs. Although not significant, our results suggest a decrease in the postoperative cost of hospital stay from $111,707 to $95,247 per patient. Lastly, we demonstrate that there was no difference in the length of the operation, change in pre- and postoperative GCS, change in ICH score, number of days spent in the hospital, and discharge disposition between both groups (all p > 0.05).

Evidence suggests that early intervention through surgery for ICH can reduce morbidity and mortality [22-24]. Gregson et al. demonstrated that early surgery for ICH, before deterioration, is beneficial, while Xia et al. showed that early hematoma evacuation can mitigate brain injury [22-24]. For MIPS, Scaggiante et al. and Xia et al. suggested that undergoing early surgery improves postoperative outcomes, decreases rebleeding rates, and improves recovery rates compared to craniotomies [15,23,24]. Although not statistically significant, our results suggest that undergoing MIPS within 12 hours of admission may reduce the mean number of days spent on a ventilator (13.8 to 9.4), the mean number of days spent in the ICU (10.4 to 5.5), the number and therefore the mean cost of postoperative CTH ($1,183 to $964), and the total mean postoperative care cost ($109,619 to $99,251). However, given the low sample size, it is difficult to assess the true magnitude of our findings.

Further research is needed to assess the effects of a leveling system in the OR. This would mean that patients with ICH who require emergency evacuation may benefit in terms of postoperative outcomes and economic burden if they undergo an operation within a shorter time after deciding whether to operate. Additionally, novel developments in artificial intelligence programs (such as Viz AI and Rapid) for the management of ICH could further reduce wait times and costs and perhaps improve postoperative outcomes since surgeons could be alerted, in real-time, of imaging results leading to an earlier decision to operate. 

Potential utility in community hospitals and areas of limited resources

As a tertiary, academic, level 1 trauma center, and Joint Commission-certified Stroke Center of Excellence, many of our patients, including those in this study, were transferred to us from rural Missouri and southern Illinois for advanced neurological care, some exceeding two hours by ground. This is not an isolated occurrence in the United States, where millions of Americans live over an hour from a level 1 trauma center and over 16% are at least 30 miles (30-40 minutes) from any medical facility, which increases morbidity and mortality in the event of medical emergencies [25]. For facilities where tertiary centers have access to radiographic studies, the ability to use the initial scans from these outside hospitals may help cut down on the time to OR in emergent transfer cases by the time required for a CT scan to be scheduled, performed, and released to the electronic medical record. “Time is brain,” and every second in these situations counts. Necessarily, avoidance of repeated imaging cuts down on the building costs for these patients, where every cent may count, as well.

In hospitals with the ability to perform surgical interventions on site, but in a medically underserved area, or in an area with a lower average socioeconomic status, this expedited protocol may be especially impactful for both the patients and the medical system. For the millions of Americans for whom there is only one medical facility within a 30-mile radius, that single medical facility may be responsible for performing a large proportion of regional outpatient imaging in addition to their inpatient responsibilities. Some hospitals report an outpatient CT scan scheduling delay of over six weeks, such that any additional imaging may cause even more delays [26].

Limitations

The limitations of this study include its small sample size, retrospective nature, and potential selection bias in the control and experimental groups. Because of our institution’s enrollment in the ENRICH trial and the difficulty of MIPS patient qualification, the sample size is limited. A significant drawback is the lack of power due to the low sample size. Although long-term conclusions are difficult to assess, this study is a pilot study and introduces the topic of reducing costs and provides a method to improve efficiency for patients undergoing MIPS for ICH evacuation. The ENRICH trial is the first surgical trial showing class I evidence regarding the superiority of surgery. The timeframe for surgery was up to 24 hours from the last known well. Our study suggests early surgery may show a trend toward further improved outcomes, but larger studies are needed within the first 24-hour window to better understand the impact of the timing of surgery. Future studies may benefit by involving multiple centers within one region to augment the sample size. The long-term impact, initial clinical course, detailed costs of in-hospital care, and assessment of comorbidities also warrant further investigation. Furthermore, we did not assess the hospital cost related to the comorbidities, as the desired outcome of this study was to understand whether reducing the number of preoperative scans does indeed reduce costs for patients with ICH. Medical comorbidities did not influence how patients were treated. The cost savings noted were most significant for a savings in imaging studies which we felt was specific to the treatment protocol used. Further studies to account for costs associated with comorbidities in patients with ICH requiring MIPS evacuation are needed to understand if comorbidities are significantly confounding the costs related to the expedited protocol, especially in the preoperative setting. We could not control the number of preoperative CTHs performed at outside hospitals, and therefore, these images were not factored into the cost calculations. The study is retrospective, making it difficult to obtain detailed functional outcomes at predetermined time points. Additionally, there is the possibility of Hawthorne’s effect in this study, as patients who underwent an expedited protocol or early surgery protocol may have had favorable preoperative and postoperative outcomes due to being observed. 

Furthermore, operative costs depend on the geographic location and institution. The OR at a large midwestern city medical center in the United States is not analogous to rural medical centers or ORs in developing countries. Analyzing the cost can indeed be challenging, as each hospital system has different billing systems that are not publicly available. Hospital billing is complex, and it is usually very difficult to assess the true cost of stay. Additionally, the cost that the hospital incurs, the cost for the insurance companies, and the cost for the patients are drastically different and very challenging to analyze. Prices and costs at our institution may vary significantly from costs at other hospital systems. Many essential hospital costs may have not been accounted for; however, the goal of this study was to show that improvements in the preoperative protocol can indeed have a positive impact on overall hospital costs by specifically reducing the number of imaging studies obtained and therefore reducing the cost of imaging without having a detrimental effect on hospital stay and short-term outcomes.

Another challenging aspect to control is the large-scale change in hospital operations and billing processes. At our institution, during this study period in 2015, our facility transitioned ownership from a publicly owned for-profit system to a private, not-for-profit system. While the impact of this change in ownership would have been delayed from the point of change, patients included later in the study period could have potentially been affected. Regarding the cost analysis aspect of this study, both academic research studies and fiscal analyses have shown that, all else considered equal, patients who receive care at for-profit facilities incur higher charges and overall costs of care than those at non-profit institutions, necessarily due to the profit motives of private, for-profit organizations. Therefore, patients who were treated after any financial changes were made as part of this transition may have seen different costs than those treated before the transition. However, as we would have no way of knowing the exact dates on which fiscal changes were officially implemented, we are unable to formally assess this change as part of this study. For future studies and larger, multi-institutional trials that may follow this study, not only should hospital ownership (for-profit vs non-profit) be considered for each included institution, but so should other potential factors, including political (i.e., healthcare and insurance policies in a given state) and socioeconomic (i.e., patients with different types of insurance), among others.

Thus, our study does not definitively represent a global standard. Despite these limitations, this is the first study to show the economic burden of preoperative protocols for MIPS and can provide a framework for future region-specific studies. As this is a pilot study, there is a need for a large multicenter, randomized study to assess the true effect of the protocols on the cost outcomes of this patient population. 

## Conclusions

Patients undergoing an expedited preoperative MIPS protocol have a statistically significant reduction in preoperative costs and a reduction in time between ED admission and surgery. The preoperative imaging costs were significantly reduced in patients who underwent an operation within 12 hours of admission. Our protocol suggests an improvement in efficiency in terms of preoperative costs. On a broader scale, our protocol provides a framework for additional centers to conduct their own cost analysis studies and contribute location-specific understanding. This study serves to introduce the topic of improving preoperative efficiency in order to save overall hospital costs.
